# Spinal Gunshot Wound With an Air Rifle: A Case Report

**DOI:** 10.7759/cureus.31597

**Published:** 2022-11-16

**Authors:** Mohammed A Alshehri, Mohammed K Almutairi, Moutasem Azzubi, Mashael S Alomari, Alwaleed K Nahhas

**Affiliations:** 1 Medical Intern, King Saud Bin Abdulaziz University for Health Sciences College of Medicine, Riyadh, SAU; 2 Pediatric Emergency Medicine, King Abdulaziz Medical City, Riyadh, SAU; 3 Pediatric Emergency Medicine, King Abdullah International Medical Research Center, Riyadh, SAU; 4 Pediatric Emergency Medicine, King Saud Bin Abdulaziz University for Health Sciences, Riyadh, SAU; 5 Neurosurgery, King Abdulaziz Medical City, Riyadh, SAU

**Keywords:** physiotherapy, surgical decompression, weakness, gunshot, spinal cord injury

## Abstract

Spinal gunshot injuries are one of the most serious injuries that can cause morbidity and mortality. We report a case of a nine-year-old boy, referred to our emergency department from another hospital, with lower limb weakness after being shot by an air gun in his back. After extensive investigation, it appeared that a foreign body representing the bullet was found in his spinal canal; the patient was taken to the operating room for surgical decompression and removal of foreign bodies. After that, the patient started to show partial improvement in his neurological deficits and is to be continued on physiotherapy.

## Introduction

Gunshot wounds have become more recognized as a direct cause of spinal cord injury [1]. The extent of the damage by a spinal gunshot wound can vary depending on the type of weapon used, the nature and diameter of the bullet, and other biological factors [2]. Gunshot injuries are classified as either civilian- or military-sustained, according to the ballistic characteristics, the impact mechanism, and the outcome [3,4]. To the best of our knowledge, there are limited studies that investigate spinal gunshot wounds that are caused by air rifles. Here we report the case of a boy that presented to our hospital with a spinal gunshot wound injury caused by an air rifle.

## Case presentation

A nine-year-old boy was referred to our emergency department by emergency medical services (EMS) after sustaining a gunshot wound in his mid-back with an air rifle. According to the father, the kids were playing with the gun and a bullet went off. He denied any history of loss of consciousness, nausea, vomiting, or head injury.

Following the incident, the patient was sent to another hospital and then referred to us on the same day for further assessment. Upon clinical examination, the patient was conscious, oriented, and actively speaking. He was afebrile, maintaining normal blood pressure, O2 saturation was 100% on room air, had a score of 15/15 on the Glasgow coma scale (GCS), and his pupils were reactive and symmetrical. Secondary survey was unremarkable apart from a small wound around 2cm to the left of the spine at the level of T12, not associated with active bleeding. There was no exit wound, and no other cut wounds. There was mild supra-pubic tenderness which was related to urine retention. Extended focused assessment with sonography in trauma (E-FAST) was negative. Upon spinal examination, the patient had intact sensation and full power in the upper extremities. However, the patient had a power of 3/5 in the left lower limb and 0/5 for the right lower limb with a positive Babinski sign, negative clonus, and negative Hoffmann sign. Upper limb reflexes were all intact, whereas the left knee reflex was the only one appreciated.

**Figure 1:**
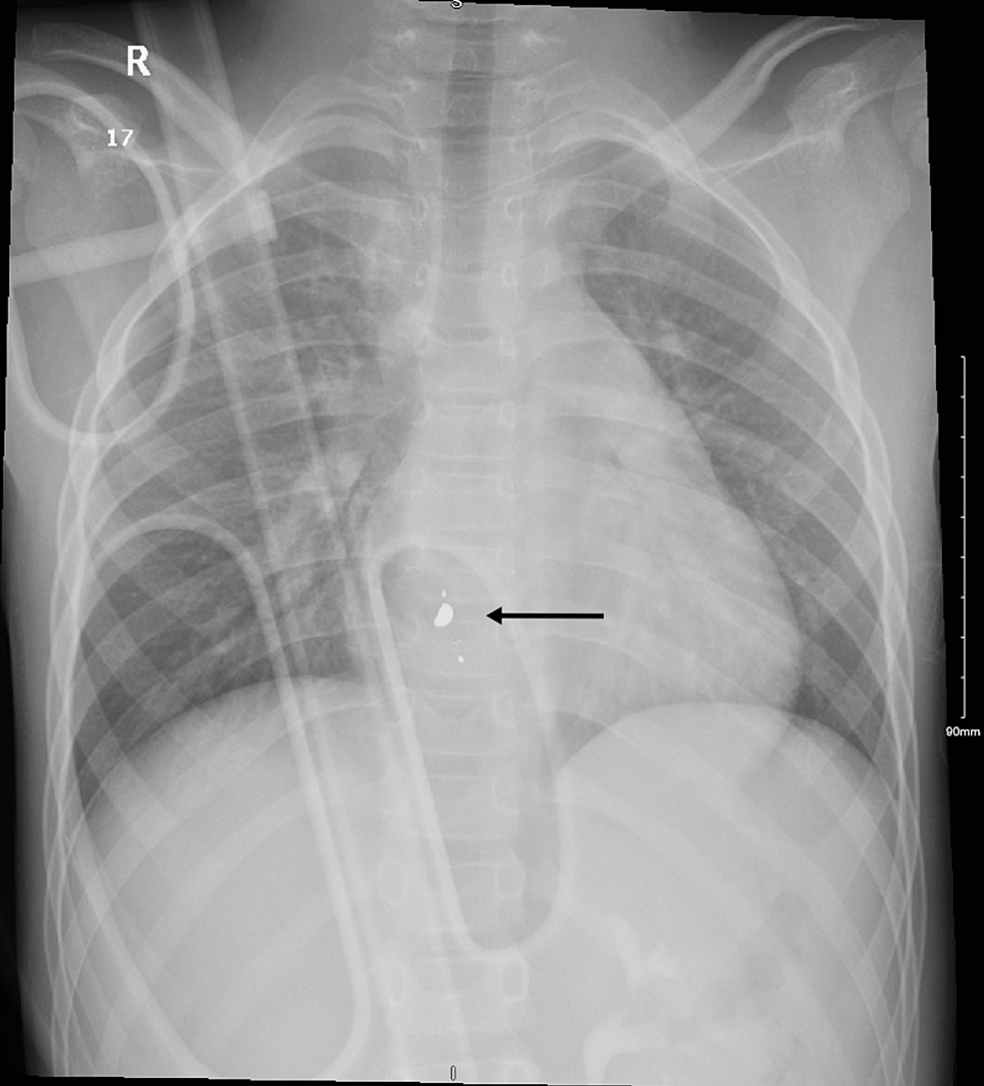
Chest X-ray showing foreign body representing the fragmented bullet.

Chest X-ray was initially ordered and it revealed a foreign body with no signs of pneumothorax or hemothorax. The trachea was centralized with no obvious fractures (Figure 1). 

**Figure 2:**
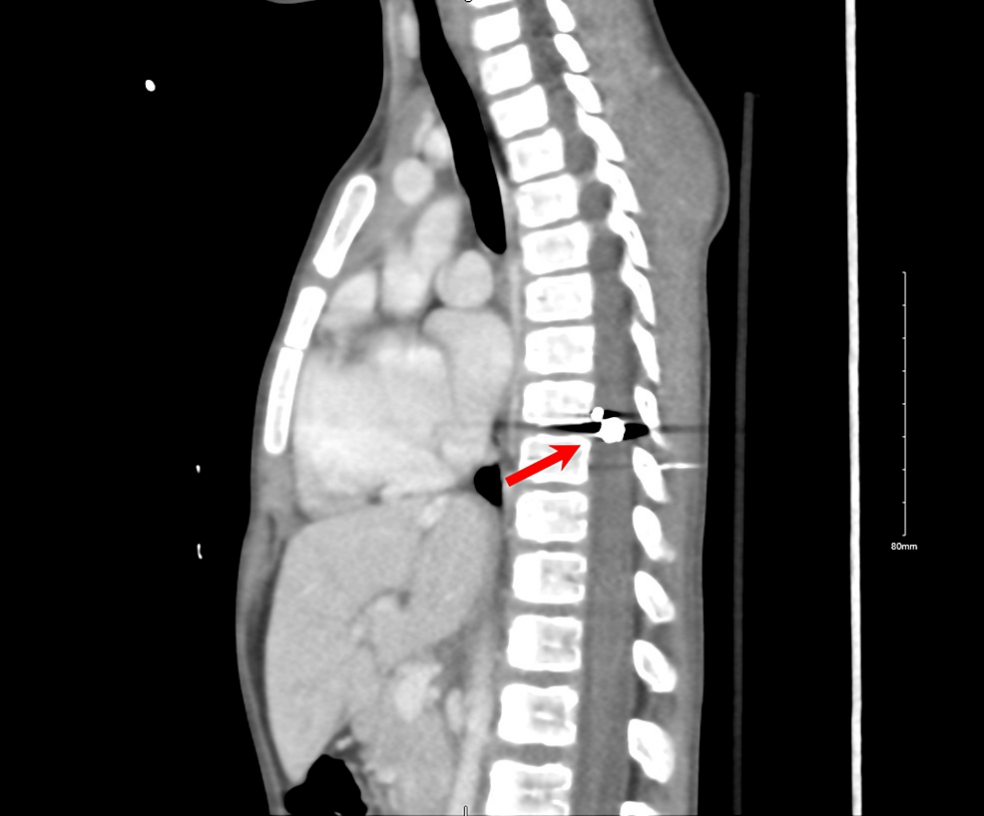
Sagittal CT scan showing the fragmented bullet at the level of T8.

CT scan confirmed a foreign body within the right lateral aspect of the spinal canal at the level of T8 representing the known bullet. Another foreign body was seen inferior to the T8 spinous process, which represents bullet fragments or is related to another bullet (Figure 2).

Therefore, the patient was planned for surgical decompression of the spine and removal of the foreign bodies after written consent from the parents. Dexamethasone and antibiotics were given prior to surgery. After induction of anesthesia, the patient was switched to the prone position. The procedure was started by performing a midline skin incision using the monopolar. The skin incision was measured around 5cm around the bullet fragment. We used portable fluoroscopy to identify the correct level of the fragmented bullet. Careful dissection was done by using the monopolar in multiple layers until identifying the spinous process, then dissection of the paraspinal muscles was done from the spinous process from both sides until identifying the spinous process. Irrigation was done through the wound. There was a clear cerebrospinal fluid (CSF) leak from the wound. We found there was a fractured bone in the left lamina of T9 which is most likely the entry site. T8-T9 laminectomy was done using a drill and Kerrison; dura was exposed, and it was found that there was a dura opening due to bullet damage. Dura was retracted using 4.30 silk. There was a clear fragment of the bullet inside the spinal canal compressing the spinal cord mainly on the right side Then we removed the big bullet and all its fragments which was also confirmed by fluoroscopy. Copious irrigation was done, then the dura was closed in a watertight fashion to prevent a CSF leak. Clean and dry dressing was applied to the closed wound. The patient was extubated and shifted to the pediatric intensive care unit (PICU) in a stable condition and was put on dexamethasone and cefazolin.

Twenty-four hours after surgery, the patient was vitally stable, afebrile, alert and oriented, with a GCS of 15/15. Upon motor examination, he was able to move all upper limbs freely with 5/5 power symmetrically. The left lower limb had a power of 2/5 in all muscle groups with normal reflexes; crude touch sensation was intact in all dermatomes as well as position sense. The right lower limb was flaccid, with absent reflexes, and intact crude sensation in all dermatomes; position sense was not intact, and power was 1/5 with distal muscles being weaker than proximal muscles. Jackson-Pratt drain (JPD) collected 65ml of CSF over 24 hours. Over the next five days, we started tapering down dexamethasone with the initiation of physiotherapy. Forty-three days after the surgery and 38 days post physiotherapy, the patient showed improvements in his neurological deficits. He was able to move his upper limbs freely, and had a power of 3/5 in all muscle groups of his left lower limb and a power of 4/5 for the right lower limb. The patient eventually was able to stand and walk a few steps with assistance and is to be continued on physiotherapy in another tertiary hospital.

## Discussion

Spinal gunshot wound injuries account for about 13% to 17% of all spinal cord injuries and are the third most common cause of spinal cord injury only after road traffic accidents and falls [5]. The most common injured areas are the thoracic area, lumbar area, and cervical area, respectively [6]. According to a review by Di Maio, a critical projectile velocity of 70 m/s is needed for an air rifle to penetrate the human skin [7]. In most cases, an accidental shooting by a friend, relative, or self-injury is involved, usually in the absence of adult supervision. Children are the most frequent victims of air gun injuries or fatalities, and the assailant is also most often a child [8]. In all fatal or non-fatal cases, boys are far more involved than girls, and the victims and assailants are most often aged 10 to 14 [9].

The treatment of spinal gunshot wounds is controversial. In military circumstances, surgery is usually indicated to prevent further damage by high-velocity guns. However, in civilian injuries, the damage is generally less due to slower projectile velocity in air rifles. Therefore, conservative management with the administration of antibiotics is the usual approach [10]. Indications for surgery include cauda/conus wounds, progressive neurological deterioration, the existence of a compressing bone fragment, the existence of a foreign body inside the spinal canal, CSF fistula, spinal instability, abscess formation, and pain syndrome in the late period. CT scan is highly sensitive for the assessment of spinal integrity and for the detection of any of the mentioned surgical indications [11]. It was evident in the study by Robertson and Simpson that complications such as radicular pain resolved after decompressive laminectomy, whereas in other studies, such as Waters and Adkins, there was no improvement even after surgical management [12,13].

## Conclusions

Spinal cord injuries are serious and sometimes fatal especially among the child population without adult supervision. Close attention while taking history and performing the clinical examination is mandatory in order to identify alarming signs and to optimize prognosis.
